# Surface Hybridization Chain Reaction of Binary Mixture DNA-PEG Corona Nanostructures Produced by Low-Volume RAFT-Mediated Photopolymerization-Induced Self-Assembly

**DOI:** 10.1021/acs.bioconjchem.3c00293

**Published:** 2023-10-16

**Authors:** Siriporn Chaimueangchuen, Jennifer Frommer, Calum T. J. Ferguson, Rachel K. O’Reilly

**Affiliations:** School of Chemistry, University of Birmingham, University Rd W, Birmingham B15 2TT, U.K.

## Abstract

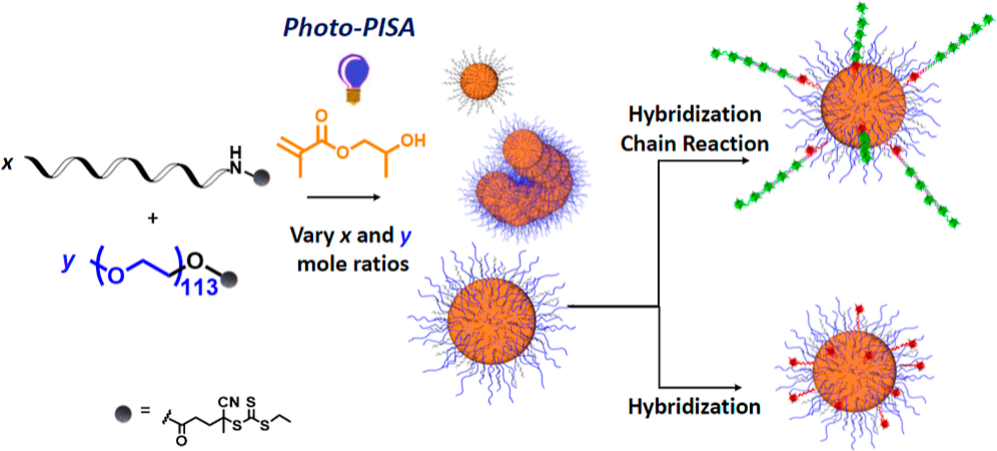

DNA-polymer hybrids have been attracting interest as adaptable functional materials by combining the stability of polymers with DNA nanotechnology. Both research fields have in common the capacity to be precise, versatile, and tunable, a prerequisite for creating powerful tools which can be easily tailored and adapted for bio-related applications. However, the conjugation of hydrophilic DNA with hydrophobic polymers remains challenging. In recent years, polymerization-induced self-assembly (PISA) has attracted significant attention for constructing nano-objects of various morphologies owing to the one-step nature of the process, creating a beneficial method for the creation of amphiphilic DNA-polymer nanostructures. This process not only allows pure DNA-polymer-based systems to be produced but also enables the mixture of other polymeric species with DNA conjugates. Here, we present the first report of a DNA-PEG corona nano-object’s synthesis without the addition of an external photoinitiator or photocatalyst via photo-PISA. Furthermore, this work shows the use of DNA-macroCTA, which was first synthesized using a solid-support method resulting in high yields, easy upscaling, and no need for HPLC purification. In addition, to the formation of DNA-polymer structures, increasing the nucleic acid loading of assemblies is of great importance. One of the most intriguing phenomena of DNA is the hybridization of single-stranded DNA with a second strand, increasing the nucleic acid content. However, hybridization of DNA in a particle corona may destabilize the nanomaterial due to the electrostatic repulsive force on the DNA corona. Here, we have investigated how changing the DNA volume fraction in hybrid DNA-polymer self-assembled material affects the morphology. Moreover, the effect of the corona composition on the stability of the system during the hybridization was studied. Additionally, the hybridization chain reaction was successfully applied as a new method to increase the amount of DNA on a DNA-based nano-object without disturbing the morphology achieving a fluorescence signal amplification.

## Introduction

DNA-polymer hybrids are adaptable functional materials with numerous applications ranging from nanotechnology to biomedicine.^1^ Both solution phase and solid support synthesis approaches have been used to create DNA-polymer hybrids.^2−5^ Unfortunately, so far, most synthetic routes have resulted in low yields, arising from the general difficulty of coupling macromolecules. Additionally, it is often challenging to find conditions compatible with both the DNA and polymer segments. Recently, to overcome these limitations the direct extension of DNA strands with polymers has been reported using reversible addition-fragmentation chain transfer-mediated polymerization-induced self-assembly (RAFT-mediated PISA) has been developed.^6^ PISA allows the direct construction of polymeric nanoparticles (NP) of various morphologies, normally spheres, vesicles, or worms.^7^ Control over particle composition, morphology, and functionality can be achieved by varying the monomer, macromolecular chain transfer agent (macroCTA), and concentration.^1^ In 2020, Luckerath et al. suggested that DNA–polymer conjugates can self-assemble to nano-objects by thermal RAFT PISA through the grafting from approach.^6^ This reaction was performed in a low volume system, relying on enzyme degassing using glucose, glucose oxidase, and sodium pyruvate to get rid of oxygen in the system which would hinder the polymerization. This resulted in high monomer conversions in a system where the monomer to initiator ratio could be precisely controlled, allowing the manipulation of architectures formed. Yang et al. modified a photochain transfer agent (photo-CTA) to perform the first aqueous RAFT photo-PISA of functional DNA-polymer nanostructures under an enzyme-assisted approach.^8^ 2-hydroxypropyl methacrylate (HPMA) was used as a monomer to manipulate the DNA-polymer nanostructure. This work showed that DNA-poly(2-hydroxypropyl methacrylate) (DNA-PHPMA) particles had enhanced nuclease resistance and increased cellular uptake.

Most natural DNA forms a double helix structure through hybridization, which is used in vivo for storing and transmitting genetic information. Hybridization is promoted by the formation of hydrogen bonds between the nucleobases of single-stranded DNA (ssDNA) with its complementary DNA (cDNA). Due to the high sequence-specificity of DNA hybridization, it is possible to construct even large and complicated DNA structures. Thus, precise DNA sequence design is the key to unlocking structure and/or sequence driven DNA-based materials in nanoscience. The advantage of an increased cellular uptake and nuclease resistance make DNA-polymer hybrids the perfect candidate for the development of a new class of a responsive biocompatible material. However, not much is known about the ability to build DNA structures in situ after DNA-polymer assembly and how DNA hybridization impacts the size and/or morphology of the DNA-polymer particles. Over the past decade, signal amplification using nucleic acids has become an attractive tool in biotechnology. Among DNA signal amplification techniques, hybridization chain reaction (HCR) is a simple yet powerful molecular tool with various applications in biosensing, bioimaging, bioanalysis, and biomedical research.^9^ The concept behind HCR is a multihybridization event between two species of DNA hairpins which are initiated by an initiator ssDNA, yielding a nicked DNA duplex with repeating units *x*. In the past, HCR products were obtained with a variety of functional moieties such as fluorophores,^10^ gold NPs,^11^ and electrochemical reagents^12^ to achieve biosensing, signal transduction, or transforming input molecules. As we know, corona modification of NPs influences the surface characteristics and properties of the nano-objects. To the best of our knowledge, HCR on DNA-polymer nano-objects has not been investigated before and represents an important step toward responsive DNA-polymer hybrids. Hence, here we aim to apply HCR to DNA-particles to prove the possibility of building larger DNA constructs on top of a short starting sequence covalently bound to the particle. The presence of the initiator DNA to gives a fluorescence read-out and signal amplification. We believe this represents not only key knowledge to achieve interactive DNA particles to act as biosensor, but moreover we show that DNA-polymers can be used as foundation for larger DNA constructs without disturbing particle morphology.

**Scheme 1:**
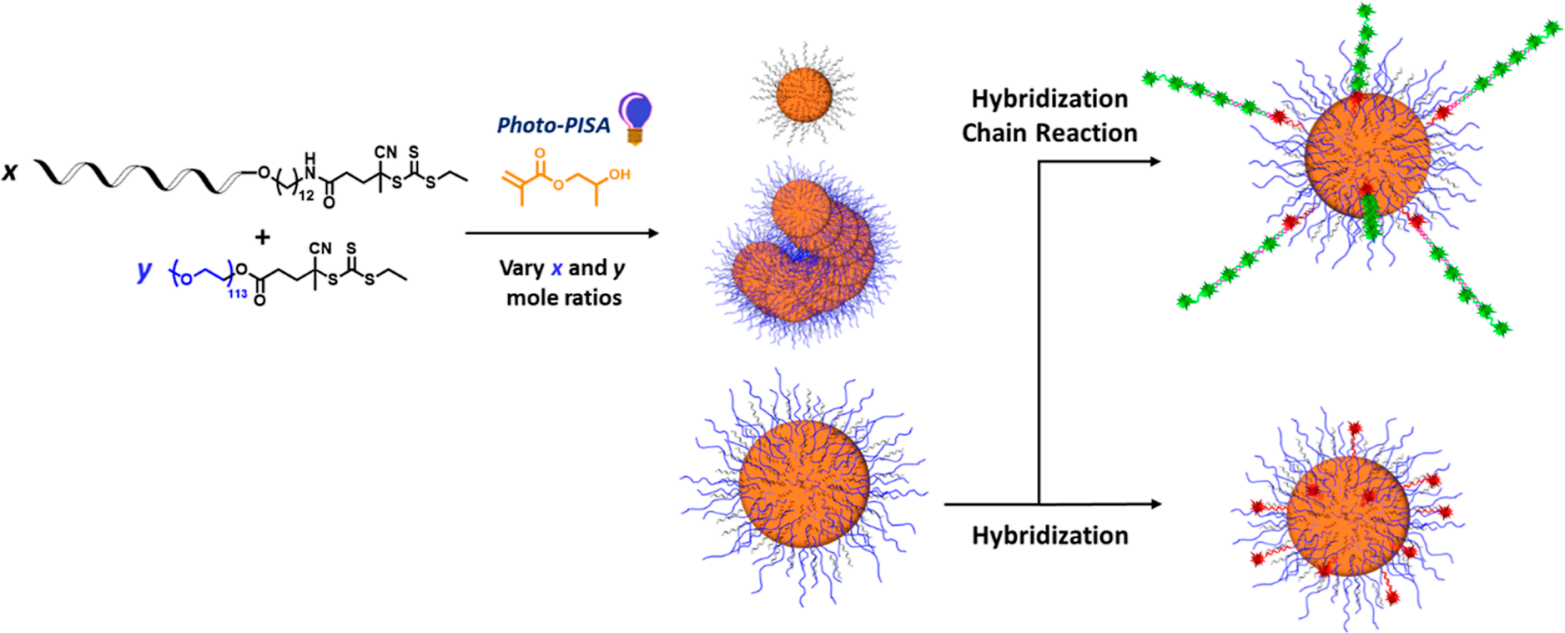
Concept for photoPISA from ssDNA_14_ or PEG_113_ CEPA-functionalized ssDNA_14_ and CEPA-PEG_113_ served as the macroCTAs in RAFT-PISA polymerization for the generation of functional DNA-copolymer conjugates. DNA–polymer nanostructures of various shapes were obtained by altering mole ratio of DNA_14_-macroCTA and PEG_113_-macroCTA, establishing a new platform technology toward functional DNA–polymer nanostructures. Moreover, hybrid vesicles were used in hybridization and HCR.

In this work, we report the first synthesis of trithiocarbonate-based ssDNA_14_-macroCTA by solid support synthesis, resulting in high yields, scalability, and high purity without the need for further high-performance liquid chromatography (HPLC) purification. This macroCTA has been combined with trithiocarbonate-based poly(ethylene glycol) (PEG) macroCTA for the novel fabrication of binary mixtures of PEG_113_-PHPMA_400_ and DNA_14_-PHPMA_400_ nanomaterials. No additionl external photoinitiator or photocatalyst was required and the synthesis was undertaken via low-volume RAFT-mediated photo-PISA using enzyme-assisted degassing. Systematic variation of the relative proportions of PEG_113_ and ssDNA_14_ macroCTAs resulted in the formation of diblock copolymer spheres, lumpy rods, or vesicles, where increasing the ratio of PEG_113_ led to the formation of higher order morphologies and more stable particles in salt/buffer solutions. Moreover, the resulting particles were further hybridized with TAMRA-cDNA. In addition, HCR was first applied to hybrid NPs to enhance the amount of DNA on the particle surface without disturbing particle morphology obtaining a fluorescence signal (Scheme 1).

## Results and Discussion

**Figure 1:**
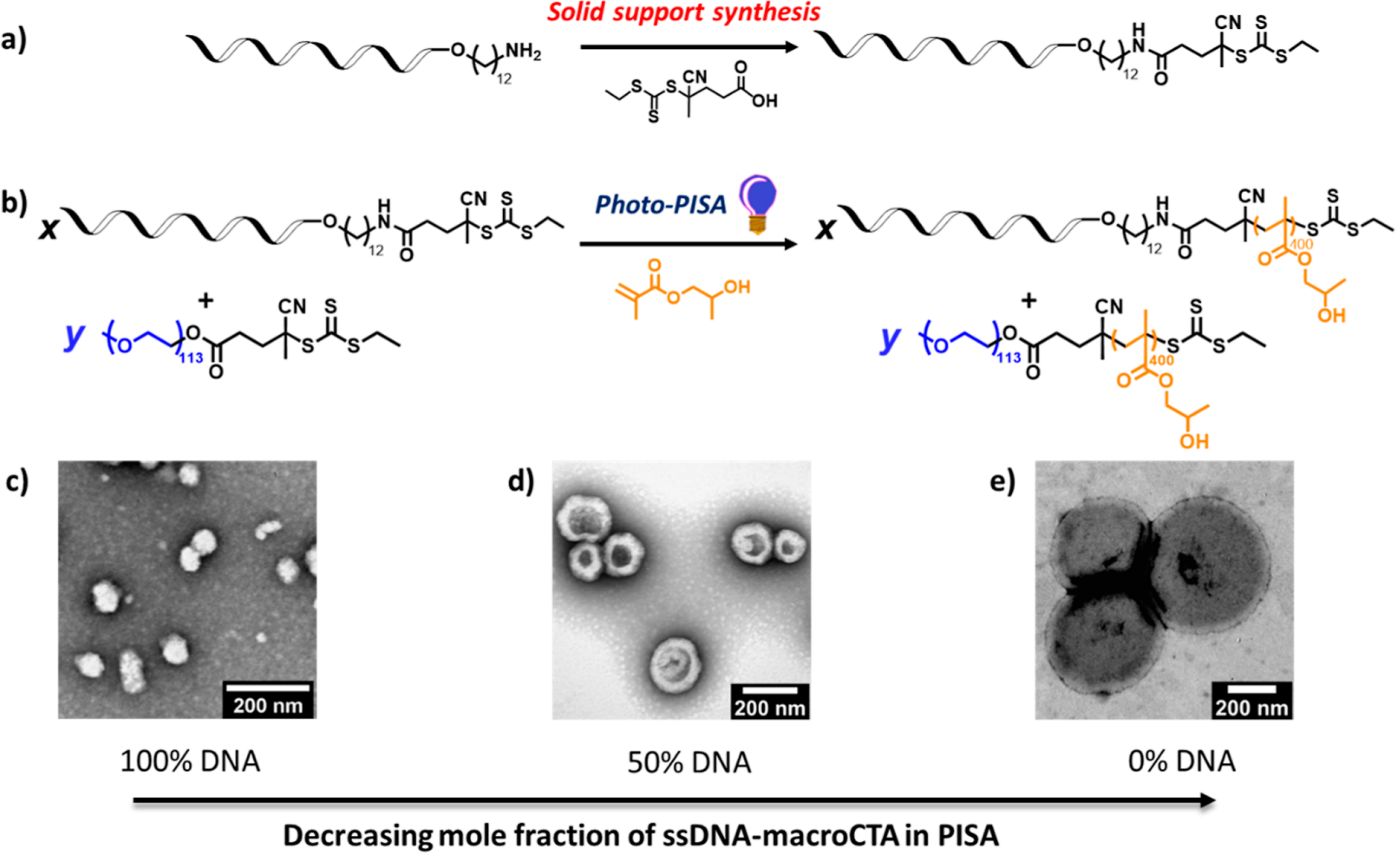
(a) Reaction scheme for the synthesis of ssDNA-macroCTAs via solid support synthesis, (b) reaction scheme for the low-volume photo-PISA for the synthesis of *x* DNA_14_–PHPMA_400_ and *y* PEG_113_–PHPMA_400_ diblock copolymer mixtures, and (c) schematic TEM images of mixed DNA and PEG based particles with different percentages of DNA in the corona.

In order to synthesize the DNA-loaded particles, a high yielding, easily scalable, and simple method for synthesizing DNA-macroCTA was needed. The ssDNA_14_ macromolecular chain transfer agent (ssDNA_14_-macroCTA) was formed by the conjugation of ssDNA_14_-NH_2_ (5′-5AmMC12-TGTAGCGTTGTTGC-3′) with a 4-cyano-4-(dodecylsulfanylthiocarbonyl) sulfanyl pentanoic acid group (CEPA) at its 5′ terminus via solution or solid support approaches (Figure 1a). By using a solid support, DNA is selectively bound to an inert solid support, i.e., diethylaminoethanol (DEAE) sepharose, followed by chemical reaction in pure *N*,*N*-dimethylformamide (DMF) and is finally eluted from the solid support without the need for further purification. The solid support-mediated synthesis is separated into four stages. During stage 1, ssDNA_14_-NH_2_ was bound to the solid support. In stage 2, the small molecule was added as activated ester to the adsorbed DNA in DMF. In stage 3, the excess small molecules including degraded side products were removed by washing with DMF, while the modified DNA remained adsorbed to the solid support. During the final stage the modified DNA was eluted using a water-based elution buffer.^13^

The purity of the ssDNA_14_-macroCTA was confirmed by using HPLC equipped with a UV–vis detector monitoring the eluted DNA solution at 260 and 309 nm as the maximum absorbance of the DNA and trithiocarbonate group, respectively. High conversion and purity (95%) of the targeted ssDNA_14_-macroCTA strand was achieved with the solid support approach, represented by the occurrence of one DNA-species signal at both detector wavelengths 260 and 309 nm (Figure S1). LC/MS furthermore confirmed the successful formation of the desired product. Meanwhile, the solution approach shows a second signal at 309 nm, indicating an impurity from the trithiocarbonate-group-containing starting material. Additional HPLC purification and extensive purification steps including spin filtration and ethanol precipitation were required to obtain a comparable purity. Such time-consuming purification steps are not feasible especially if high amounts of ssDNA_14_-macroCTA are required (Figure S1). Our results show that the solid support-mediated synthesis of the desired macroCTA-modified DNA is not only superior in terms of yield and purity but is moreover easy to apply to large-scale DNA reactions (up to 100 nmol) without the need of further purification, while the solution method is limited to 2 nmol DNA in each reaction batch. Thus, we accomplished the synthesis of the ssDNA_14_-macroCTA in a few hours with the large quantities, high yields, and purity, needed for further utilization in a DNA nano-object PISA reaction.

A series of PEG_113_-PHPMA_400_ and DNA_14_-PHPMA_400_ diblock copolymer NPs were synthesized by systematically varying the PEG_113_ and ssDNA_14_ macroCTAs mole fractions via low-volume RAFT-mediated photo-PISA. These CTAs are photoiniferters, which can be cleaved by light at 405 nm to generate a thiocarbonylthio radical.^14−16^ This radical subsequently serves the dual roles of initiating the polymerization of a monomer and acting as a RAFT agent, eliminating the requirement for additional initiators or catalysts. Thus, this is the first report of DNA-polymer nanostructures produced via low-volume PISA (50 μL) without adding an external photoinitiator or photocatalyst. PEG_113_-macroCTA was chosen due to its biocompatible properties being extensively studied *in vivo* to protect DNA from degradation, and its similar molecular weight to ssDNA_14_-macroCTA.^17^ HPMA was chosen due to the literature precedent for forming a hydrophobic polymer block with hydrophilic polymer chain such as PEG.^18^ Low-volume aqueous RAFT-mediated photo-PISA of HPMA was undertaken under 405 nm visible light irradiation at 37 °C using an enzyme degassing approach, i.e., glucose oxidase.^19,20^ Mineral oil was added on top of the 50 μL solution to prevent diffusion of residual oxygen into the solution, which inhibits the polymerization.

Table S1 summarizes the targeted diblock copolymer compositions, HPMA monomer conversions, molecular weight data, dynamic light scattering (DLS) data, and morphological assignments. High monomer conversions (>70%) were achieved after 2 h of reaction, as determined by ^1^H NMR spectroscopy. It is important to note that the targeted degree of polymerization for PHPMA is 400. However, incomplete polymerization conversion might have an effect on the resulting morphology of the self-assembled nanostructure. PHPMA was successfully polymerized with a significant increase in molecular weight confirmed by size exclusion chromatography analysis (in DMF) (Figure S2). Zeta potentials have been applied to measure the surface electrical charge of particles. The zeta potential of 100% DNA-PHPMA_400_ was found to be around −32.4 ± 9.7 mV revealing the presence of negative charge from DNA on the outer surface of particles, whereas pure PEG_113_-PHPMA_400_ exhibited a nearly neutral surface charge (−0.6 ± 3.1 mV). The 50% DNA-PHPMA_400_ displayed a negative charge of approximately −28.6 ± 7.0 mV, which falls between the values of the 100% DNA and PEG_113_-PHPMA_400_ systems. This rationally indicated that the negative charge from DNA on the particle surface decreases when the fraction of DNA is reduced.

The morphology of the self-assembled nanostructures was determined by transmission electron microscopy (TEM) and cryogenic TEM (Cryo-TEM) images of each diblock composition was used to assign the copolymer morphology, as shown in Figures 1c–e and S3, respectively. When 100% ssDNA_14_–PHPMA_400_ diblock copolymer chains self-assembled only spherical particles formed (Figure 1c). This morphology is most likely due to the strong repulsive electrostatic forces of the DNA block on the corona, as also previously reported with anionic corona of block copolymers.^21^ When 90% of the DNA based diblock copolymer was used, a mixture of spheres, lumpy rods, and vesicles was observed (Figure S4a). Interestingly, for all systems with 50% DNA copolymer or less DNA content, only vesicles were observed. This morphology transition was observed because increasing the mole fraction of the PEG_113_-macroCTA reduces the electrostatic repulsion from DNA chains in the corona. Thus, as the proportion of the PEG_113_-macroCTA is increased, the volume fraction of electrostatic chains in the coronal layer is gradually reduced, which means that the volume fraction of the hydrophobic PHPMA block required to access the lumpy rods or vesicles is correspondingly lower. DLS was used to measure the hydrodynamic diameter (*D*_h_), where the 100% ssDNA_14_–PHPMA_400_ copolymer displayed a unimodal particle size distribution with *D*_h_ approximately 90 nm (Figure S5a), the 90% DNA system had a multimodal particles size distribution (Figure S5b). In the 50, 10, and 0% DNA systems, unimodal particle size distributions were observed with the *D*_h_ increasing from 150 to 460 nm as the DNA content decreased (Table S1 and Figure S5c–e). This increase in hydrodynamic diameter is consistent with the formation of higher order polymeric morphologies, as the bilayer structure of the vesicles increases the size of the nanomaterial.

**Figure 2:**
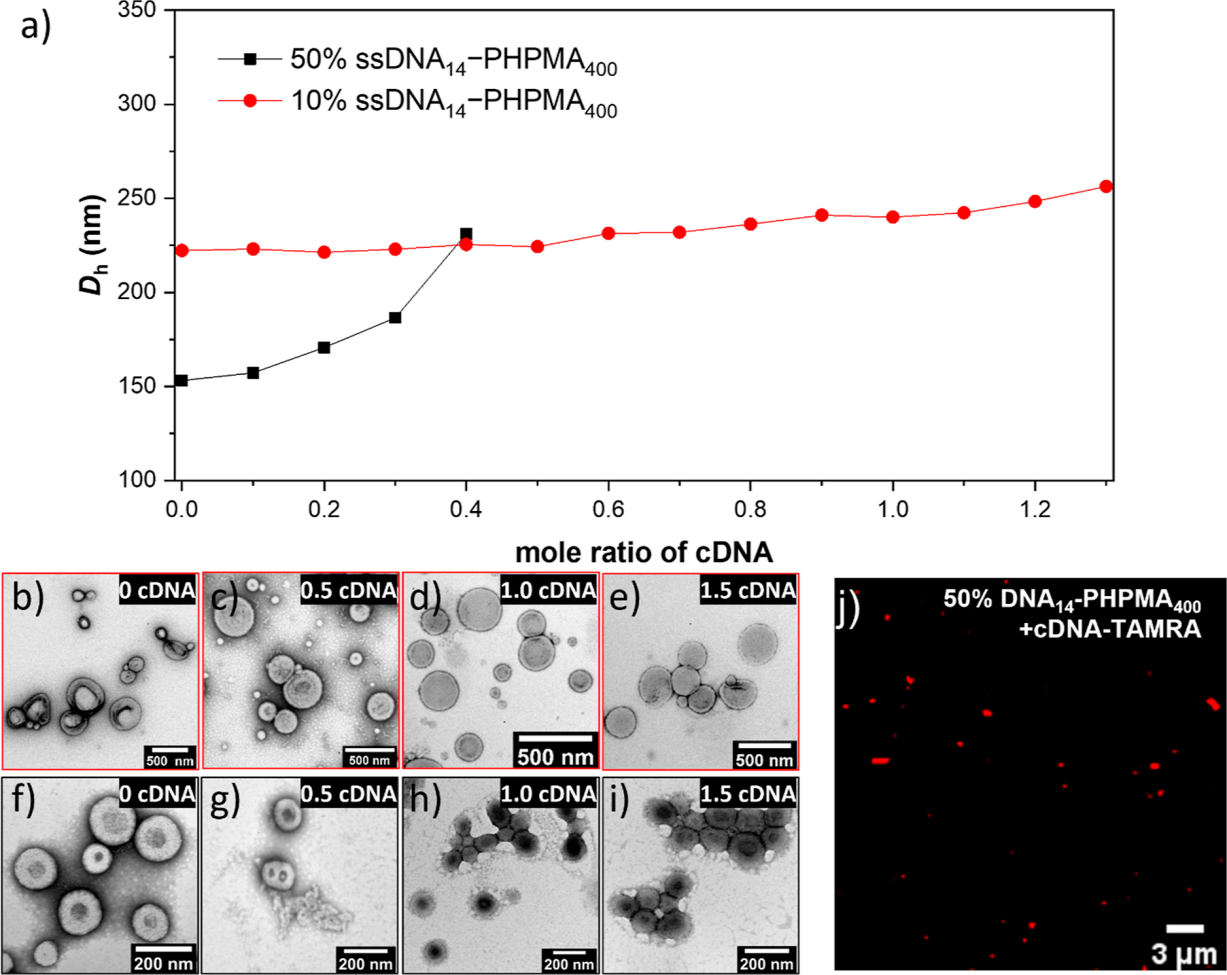
Hybridization study of DNA containing NPs. (a) Hydrodynamic radius of 50% (black) and 10% (red) DNA containing particles measured by DLS with the increase in quantity of additional DNA added. (b–e) TEM images of 10% DNA containing structures with the increase in additional DNA content. (f–i) TEM images of 50% DNA containing structures with the increase in additional DNA content. (j) Confocal images in the fluorescent field. Scale bar: 3 μm. Images are shown for 50% DNA corona NPs mixed with cDNA-functionalized TAMRA.

DNA hybridization technology allows for the postmodification of DNA due to its intrinsic functional property. In this work, DLS was used to primarily investigate the hybridization of ssDNA on different NPs. A salt/buffer solution is required to study hybridization in order to screen the effect of DNA. When 100 and 90% DNA corona composition systems were investigated, the DLS could not detect particles in salt/buffered solution due to aggregation as confirmed by TEM images. This may be due to the influence of magnesium ions (Mg^2+^) from the salt solution on the self-assembly process (Figure S6).^22^ As expected, when the mole fraction of DNA on particles decreased, the screening effect had less of an effect, and particles are more stable. Subsequently, the DNA functionality for hybridization of NPs with varying DNA content (50 and 10%) was probed by DLS using a complementary DNA strand (cDNA) (5′-GCA ACA ACG CTA CA-3). For NPs with 50% DNA, the size of particles gradually increased until a cDNA ratio = 0.4 at which point the quality of the DLS data deteriorated, indicating aggregation as also confirmed by TEM (Figure 2f–i). At the decreased volume fraction of DNA corona of 10%, the NPs were more stable when adding cDNA, until a 1.2 mol-ratio where aggregation was observed (Figure 2a). PEG chains are uncharged, and thus, positively charged salts in solution cannot interact with the PEG chains, lowering the cross-system interaction between the negatively charged DNA strands and positively charged salts which are very likely responsible for aggregation. Additionally, when the mole fraction of DNA in the corona is decreased, the DNA density on the particle surface is also decreased, while the PEG density is higher, leading to more flexible space on the particle’s surface. Thus, the relative amount of cDNA that can hybridize with the DNA in the particle corona increases when a decreasing DNA mole fraction is used. This results in relatively more stable particles due to less charge repulsion and salt interactions.

To confirm that hybridization of cDNA was occurring on the surface of the NPs, confocal fluorescence microscopy was used; this method directly images the fluorescent cDNA-particles hybridized on the DNA-corona which means the turbidity or the effect from photobleaching of fluorophores is negated. The 50% DNA system was selected to study hybridization with fluorescent TAMRA-cDNA by confocal fluorescence microscopy, as it has a high DNA mole fraction but is still stable. A mixture of TAMRA-cDNA and DNA containing NPs were clearly observed as stable dots in the fluorescence images (Figure 2j). Conversely, a mixture of cDNA and ssDNA, as a control, resulted in fluorescent specks being observed, which can happen when dyed molecules aggregate (Figure S7a). For DNA containing NPs alone, particles had no observable fluorescence (Figure S7b). To confirm that cDNA was not entangled but hybridized on the surface of DNA-polymer particles, we mixed PEG_113_-*b*-PHPMA_400_ with TAMRA-cDNA. PEG_113_-*b*-PHPMA_400_ particle were not observed in the fluorescent field; therefore confirming that there is no interaction between PEG and cDNA (Figure S7c). Thus, confocal imaging confirmed the presence of DNA at the particle surface and its ability to hybridize with a complementary strand. This means DNA-polymer particles can simply be functionalized after the assembly process by adding fluorophore-labeled cDNA with a maximum 0.4:1 mol ratio of cDNA to the 50% DNA corona containing NPs. However, if greater loadings of nucleic acid are required for larger DNA constructs the system becomes destabilized and alternative strategies are required.

To quantify the amount of DNA that could be functionalized on each particle, static light scattering (SLS) was used to obtain aggregation numbers (*N*_agg_) for the 50% DNA corona NPs. Here, for this mixed DNA and PEG based system, each particle consists of approximately 2700 chains (see Figure S8). Therefore, DNA constitutes about 1350 chains per particle. From the hybridization study, approximately 500 cDNA chains were able to hybridize with DNA on the surface of particle without negatively impacting the colloidal stability of the system. This means that less than half of the DNA chains on the surface of the particle can be hybridized before the system destabilizes.

**Figure 3:**
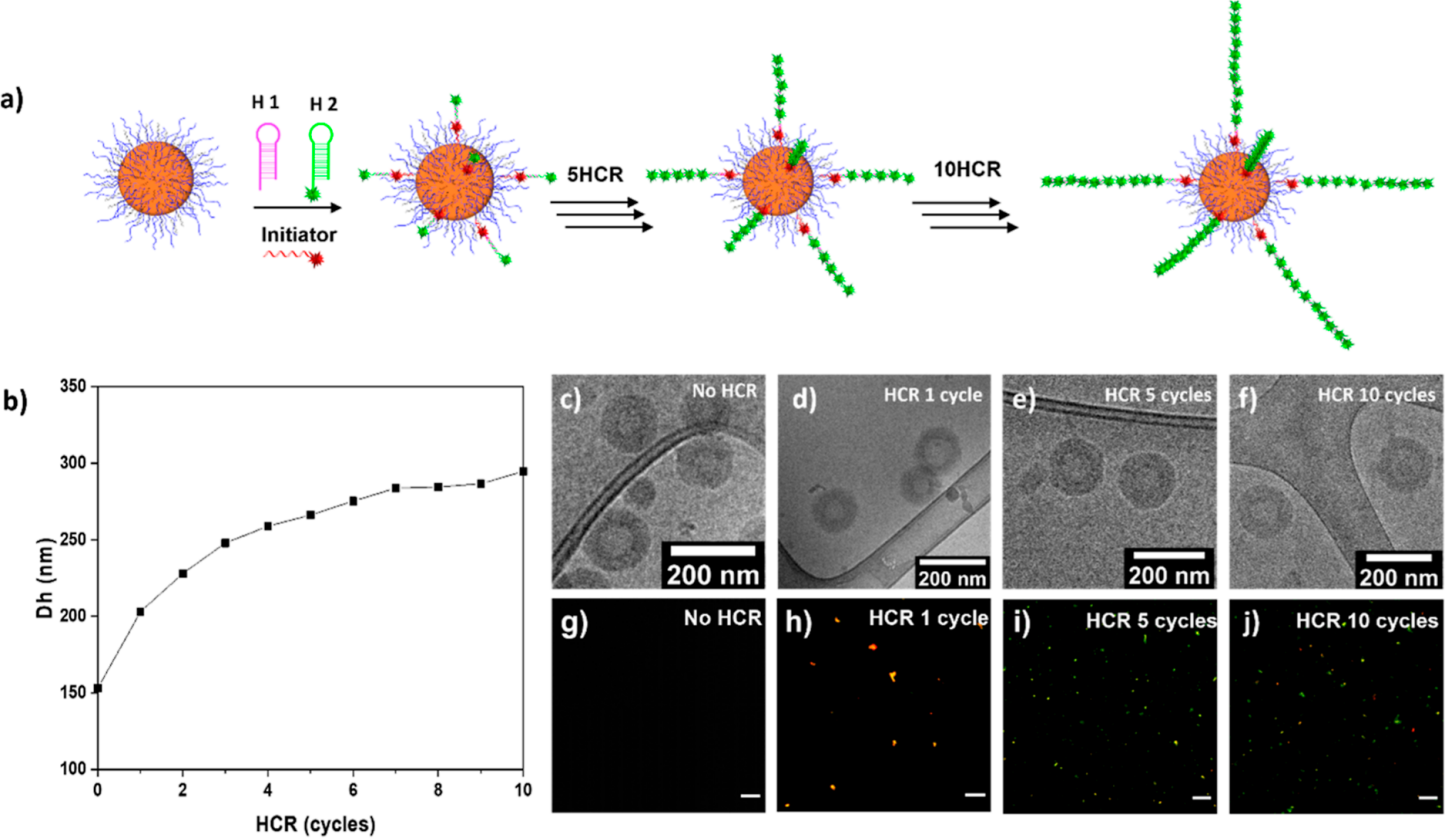
HCR study of 50% DNA corona NPs particles. (a) Schematic cartoon of HCR on particle. (b) Hydrodynamic radius of particles measured by DLS. (c–f) Cryo-TEM images. Scale bar: 200 nm. (g–j) Overlay of confocal images acquired using the 488 and 561 nm excitation channels. Scale bars: 5 μm. Images are shown for (c,g) 50% DNA corona NPs, (d,h) particles at the HCR 1 cycle, (e,i) particles at the HCR 5 cycle, and (f,j) particles at the HCR 10 cycle.

To increase the amount of nucleic acid loading on the particle corona, we investigated using HCR to increase DNA content while also producing a fluorescence read-out signal, which is desired for the adaptation of DNA-polymers as biosensors. HCR is an interesting approach because of its enzyme-free nature, use of isothermal conditions, uncomplicated protocols, and exceptional amplification efficiency.^9^ Furthermore, the initiator strand can be seen as a target sequence for future biosensor applications. Again we investigated using a 50% DNA corona NP as it is stable in salt/buffer conditions and shows vesicle nanostructure which could be investigated by confocal microscopy using fluorescently labeled DNA hybridization technology. HCR was carried out using the ssDNA_14_ chain on the particle surface by hybridization with the 50mer initiator sequence (**I**) in step 1, followed by the addition of hairpin 1 (**H1**) and hairpin 2 (**H2**) representing 1 cycle of HCR (Figure 3a), whereas the DNA sequences are shown in Table S2. Initiator **I** was added with a ratio of 0.2 relative to the ssDNA covalently bound on the particle surface to ensure that all of **I** hybridized with the particle surface, and the particles do not form aggregates. It should be noted that the two hairpin species (**H1** and **H2**) can only hybridize in the presence of the initiator DNA (**I**) on the particle surface (Figure S8) noting that only the one addition of **I** is necessary. To increase the number of performed HCR cycles, **H1** and **H2** were added stepwise up to 10 cycles, forming linear duplex DNA chains on the particle surface. Successful hybridization on the toehold of the added hairpin triggers the unfolding or opening of the hairpin structure revealing the next available single stranded DNA for hybridization in a staggered pattern. HCR study was investigated by DLS, Cryo-TEM, and confocal fluorescence microscopy. DLS data show *D*_h_ increase significantly from approximately 150 to 300 nm (Figure 3b). Interestingly, Cryo-TEM images show that the particle size does not significantly change. The distinction between these outcomes arises from the fundamental disparity in how the two techniques quantify particle dimensions. Since the diameter of double-helix DNA is approximately 2 nm, Cryo-TEM primarily images the size of the phase-separated core material, while it is unable to visualize the solvated corona due to limitations in machine resolution. Consequently, the size of the particles remained constant when the HCR process was employed to extend the corona. In contrast, DLS determines the *D*_h_ of particles, measuring not only the particle core size but also any surface structure or solvated corona. Thus, *D*_h_ undergoes a significant change when the corona length is extended through the HCR process. Moreover, this evidence suggested that the stability of 50% DNA-PHPMA_400_ NPs was maintained even when a substantial amount of DNA was added to their surface. To confirm successful multicycle HCR on the particle surface, TAMRA-labeled initiator (TAMRA-**I**) and FAM-labeled hairpin 2 (FAM-**H2**) were used and investigated by confocal fluorescent spectroscopy in merged fluorescent channel. As expected, the starting material particles did not show any fluorescence (Figure 3g). After the addition of TAMRA-**I** the particles show a red fluorescence from the TAMRA dye due to hybridization between TAMRA-**I** with the particles (Figure S9). Particle fluorescence changed to yellow when **H1** and FAM-**H2** were hybridized to particle-**I** after 1 HCR cycle caused by a combination of the fluorescence emission from TAMRA-**I** (λ_em_: 580 nm) with the fluorescence emission from FAM-**H2** (λ_em_: 517 nm) at an equal ratio (Figure 3h). When the number of HCR cycles increased, the particles exhibit higher fractions of green fluorescence due to increased ratios of FAM-**H2** (Figure 3i,j). The relative fluorescence intensity in the 488 nm excitation channel versus HCR cycles is reported in Figure S10; fluorescence values were calculated for each particle from the CLSM images in Figure 3g–j. The data showed an increase in fluorescence intensity as the HCR cycles increased, providing quantitative confirmation of the extension of DNA chains on the DNA-polymer NPs and a successful fluorescence signal amplification. Hence, this confirms that DNA-polymer particles were successfully functionalized on its surface by HCR for up to 10 cycles without aggregation and disturbing particle morphology. To predict the quantitative DNA chains on particle that are able to form HCR, SLS data were applied to investigate if ∼270 DNA strands per particle were able to form HCR.

## Conclusions

In conclusion, we have introduced the first synthesis of trithiocarbonate-based ssDNA_14_-macroCTA by solid support synthesis, resulting in high yields, easy upscaling, and purity without the need for further purification. With the use of low-volume RAFT-mediated photo-PISA under enzyme-assisted degassing conditions, this ssDNA_14_-macroCTA has been combined with trithiocarbonate-based PEG_113_-macroCTA to fabricate the first mixture of PEG and a DNA hybrid. Crucially, the particles were further applied with hybridization and HCR techniques with the simple addition of complementary DNA strands. This provides a convenient route for the synthesis of ssDNA-macroCTA, performing simple PISA in the construction of complex DNA–PEG polymer architectures, and straightforward applications with hybridization for bioimaging. HCR was first applied with DNA–PEG polymer architectures as a tool obtaining a large DNA construct on the particle surface without disturbing the particle’s morphology while producing a fluorescence read-out. These methods could address the challenges of applying DNA nanotechnology to engineer on polymer surfaces for constructing responsive nanomaterials.

## Supporting Information Available

Experimental section, additional characterization of the DNA based nanoobjects, and details on the hybridization of the DNA-based structures (PDF)

## Abbreviations

cDNA: complementary DNA
CEPA: 4-cyano-4-(dodecylsulfanylthiocarbonyl) sulfanyl pentanoic acid
DNA: deoxyribonucleic acid
HCR: hybridization chain reaction
HPLC: high-performance liquid chromatography
HPMA: 2-hydroxypropyl methacrylate
macroCTA: macromolecular chain transfer agent
PEG: poly(ethylene glycol)
PHPMA: poly(2-hydroxypropyl methacrylate)
RAFT-mediated PISA: reversible addition fragmentation chain transfer mediated polymerization-induced self-assembly
ssDNA: single-stranded DNA
