# Supplementary material for: Surface Hybridization Chain Reaction of Binary Mixture DNA-PEG Corona Nanostructures Produced by Low-Volume RAFT-Mediated Photopolymerization-Induced Self-Assembly
Source: Bioconjug Chem. 2023 Oct 16;34(11):2007–13. doi: 10.1021/acs.bioconjchem.3c00293 (PMC10655036; doi:10.1021/acs.bioconjchem.3c00293)
Supplement: Supplementary file 1 — bc3c00293_si_001.pdf [file bc3c00293_si_001.pdf]

Supporting Information

Surface hybridization chain reaction of binary mixture DNA-PEG corona nanostructures produced by low-volume RAFT-mediated photopolymerization-induced self-assembly.

Siriporn Chaimueangchuen,[†] Jennifer Frommer,[†] Calum T. J. Ferguson,[†] and Rachel K. O'Reilly[*,†]

[†] School of Chemistry, University of Birmingham, Edgbaston, Birmingham, B15 2TT, UK

*Corresponding Authors: r.oreilly@bham.ac.uk (R.K.O.R.)

Experimental Procedure

Chemicals

Poly(ethylene glycol) methyl ether (average Mn=5,000 g mol-1, PEG-OH), carbon disulfide (anhydrous, ≥99%), glucose oxidase from Aspergillus niger (100 000–250 000 U/g), sodium ethanethiolate, N,N′-dicyclohexylcarbodiimide (99%, DCC), 4-(dimethylamino)pyridine (≥98%, DMAP), were purchased from Sigma Aldrich and used without further purification. Iodine, D-Glucose, Diethyl ether, dichloromethane (DCM), and Magnesium chloride ($MgCl_2$) were purchased from Fisher Scientific. Ethyl acetate was purchased from VWR Chemicals. 2-Hydroxypropyl methacrylate (mixture of isomers, 98%, HPMA) was purchased from Alfa Aesar and was passed through a column of basic alumina to remove inhibitor prior to use and stored at 4 °C. Dry solvents used in the experiments were obtained by passing over a column of activated alumina using an Innovative Technologies solvent purification system. Formvar-coated copper grids were purchased from EM Resolutions. SYBR™ Gold Nucleic acid gel stain (10,000X concentrate in DMSO) was purchased from ThermoFisher. Tris-acetate-EDTA (TAE) buffer was purchased from Sigma-Aldrich and contains 0.4 M Trisacetate and 0.01 M EDTA. Oligonucleotides were purchased from Integrated DNA technologies, Inc. and resuspended in 18 MΩ H_2O to a concentration of 100 µM or 1 mM before use. Concentrations were calculated from the absorbance values at 260 nm using the reported extinction coefficients.

Methods

^{1}H-NMR spectra were recorded at 400 MHz on a Bruker DPX-400 spectrometer using methanol-*d4* (CD_3OD) as the solvent. Chemical shifts of protons are reported as δ in parts per million (ppm) and are relative to tetramethylsilane (TMS) at $\delta = 0$ ppm when using solvent residual peak (CH_3OH, $\delta = 3.31$ ppm).

Reversed Phase High Performance Liquid Chromatography (RP-HPLC) analysis of oligonucleotides was performed on a modular Shimadzu instrument with the following modules: CBM-20A system controller, LC-20AD solvent deliver module, SIL-20AC HT autosampler, CTO-20AC column oven, SPD-M20A photodiode array UV-Vis detector, RF-20A spectrofluorometric detector and a FRC-10 fraction collector. Chromatography was performed on a Waters XBridge™ OST C18 2.5 µM column heated to 60 °C. Flow rate was set at 0.8 mL min^{-1} using buffers A and B: buffer A, 0.1 M triethylammonium acetate (TEAA, pH 7.0), in a 95:5 mixture of H_2O and acetonitrile; buffer B, 0.1 M TEAA (pH 7),

30:70 mixture of H_2O and acetonitrile. The buffer gradient for analysis and purification was 1% buffer B for 5 minutes, 1% to 30% B over 15 minutes, 30% to 95% B over 5 min, 95% to 1% B over 1 min and finally 1% B for 3 min.

Liquid Chromatography-Mass Spectrometry (LC-MS) analysis of oligonucleotides was performed on an Agilent 1200 HPLC system coupled to a Bruker AmazonX high resolution ion trap, in negative ion mode. The desalted oligonucleotide samples were eluted through a XBridge oligonucleotide BEH C18 column (130 Å, 2.5 μm, 4.6 x 50 mm) using a 5 vol% MeOH, 10 mM ammonium acetate (buffer A) and a 70 vol% MeOH, 10 mM ammonium acetate (buffer B) solvent system at 0.8 mL/min flow. The data was processed using Compass Data Analysis (Bruker) v.4.1 software, and the MaxEnt integrated deconvolution algorithm. Alternatively, LCMS analysis was performed on a Waters ACQUITY UPLC system coupled to a Xevo G2-XS QToF mass spectrometer in negative ion mode. The oligonucleotides were eluted through an AQUITY UPLC oligonucleotide BEH C18 column (130Å, 1.7 μm, 2.1 x 50 mm) using a 75 mM triethylammonium acetate (TEAA, pH 7.0) solution in H_2O (buffer A) and a 75 mM TEAA solution in MeCN (buffer B) at 60 °C and a 0.2 mL/min flow. Leucine enkephalin was used as the reference for the LockSpray correction. The raw continuum data was deconvoluted to produce zero charge mass spectra using ProMass HR for MassLynx (Novatia) software.

Gel Electrophoresis - Native polyacrylamide gel (10%) was prepared by mixing 2.5 mL 30% 29:1 acrylamide:bisacrylamide, 4.25 mL H_2O, 0.75 mL 10×TAE, 75 μL 10% (w/v) ammonium persulfate (APS), and 7.5 μL tetramethylethylenediamine (TEMED). Different percentage gels were prepared by varying the proportions of acrylamide and water. After removing well combs, wells were rinsed with running buffer (1×TAE) using a pipette before loading 2 μL samples typically diluted to 200 nM in loading buffer (1×TAE, 50% glycerol). Native polyacrylamide gels were run at room temperature in 1×TAE buffer at 180 V using a vertical nucleic acid electrophoresis cell connected to a PowerPack basic power supply (BioRad). Samples were combined with 20% loading buffer (0.05% bromophenol blue, 25% glycerol, 1x TAE) prior to running. Non-fluorescent DNA was stained using a 1:1000 aqueous SYBR® Gold nucleic acid gel stain (ThermoFisher) and visualized using a BioRad ChemiDoc™ MP Imaging system. The images were processed using ImageLab software v 6.0.1.

1 × TAE buffer consisted of 40 mM Tris-acetate and 1 mM EDTA. 1 × TAE buffer consisted of 10 mM Tris-HCl and 1 mM EDTA. The native loading buffer consisted of 25 % glycerol and 0.05 % bromophenol blue in 1 × TAE buffer, and was diluted five-fold before use.

Size exclusion chromatography (SEC) analysis was performed on a system composed of a Varian 390-LC-Multi detector suite equipped with a Varian Polymer Laboratories guard column (PLGel 5 μM, 50 × 7.5 mm), two Mixed-C Varian Polymer Laboratories columns (PLGel 5 μM, 300 × 7.5 mm) and a PLAST RT auto-sampler. Detection was conducted using a differential refractive index (RI) and an ultraviolet (UV) detector set to λ = 309 nm. The mobile phase used was DMF (HPLC grade) containing 5 mM NH4BF4 at 50 ˚C at a flow rate of 1.0 mL min-1. Poly(methyl methacrylate) (PMMA) standards were used for calibration. Molecular weights and dispersities were determined using Cirrus v3.3 SEC software.

Zeta potential was measured by the technique of microelectrophoresis, using a Malvern Zetasizer Nano ZS instrument, at room temperature at 633 nm. All reported zeta potential values were the average of at least three runs with at least 40 measurements recorded for 3 runs. Zeta potential was calculated from the corresponding electrophoretic mobilities (μE) by using the Henry's correction of the Smoluchowski equation (μE = 4π ε0 εr ζ (1+κr)/6π μ).

Hydrodynamic diameters (D_h) of particles were determined by dynamic light scattering (DLS) using a Malvern Zetasizer Nano ZS with a 4 mW He-Ne 633 nm laser module operating at 25 ^{o}C. Measurements were carried out at an angle of 173° (back scattering), and results were analyzed using Malvern DTS 7.03 software. All determinations were repeated 4 times with at least 10 measurements recorded for each run. D_h values were calculated using the Stokes-Einstein equation where particles are assumed to be spherical, while for cylindrical particles DLS was used to detect multiple populations and obtain dispersity information.

Static Light Scattering (SLS). For the particles in deionized water, light scattering data was collected over the whole angular range, 30 < θ < 50° with the sample maintained at 25°C. Autocorrelation functions calculated by the ALV LSE-5004 correlator unit were recorded at each (θ, c) and the REPES algorithm was used to determine relaxation times, τ(θ, c). The data set τ(θ, c) was then analyzed to estimate the mean translational diffusion coefficient according to the Stokes-Einstein equation. An empirical measurement was made of the refractive index increment for the polymer in deionized water using a differential

refractometer, model DnDc1260 supplied by PSS GmbH. The light scattering experiments were conducted at 0.01, 0.02, 0.05, 0.10 and 0.20 mg·mL^{-1} to account for concentration effects. Following Andersson *et al.*[1], a Zimm plot was constructed using the Debye method (**Equation 1** and **Equation 2**) to determine the R_g of the nanoparticles. To do this, the R_θ/Kc versus q^2 data were plotted and a third order polynomial model was used to extrapolate $q \rightarrow 0$. The fit's intercept provided the molecular mass according to light scattering (M_{LS}) while the slope at $q^2 = 0$ can be utilized to retrieve R_g at the different concentrations. A first order model was utilized for the $c \rightarrow 0$ extrapolation yielding R_g and M_{LS} (Table S5).

$$\frac{R\theta}{Kc} = -\frac{MRg^2}{3}q^2 + M \quad (1)$$

$$\frac{\partial\, R\theta/Kc}{\partial q^2} = -\frac{MRg^2}{3} \quad (2)$$

The mean translational diffusion coefficient (D_{app}) was calculated from the relaxation times at each angle, $\tau(\theta, c)$ determined from the autocorrelation functions at each angle (θ, c) by the REPES algorithm. The Stokes-Einstein equation was used to determine the hydrodynamic radius (R_h) of the particles. The R_g/R_h ratio gives information about the inside of the spherical particle. A value of 1 indicates a hollow sphere with all the mass in the outer shell (i.e. vesicle). The 50%DNA-PHPMA$_{400}$ nanoparticles have a R_g/R_h ratio of 1.02. The aggregation number ($M_{w,theo}/M_{LS}$) was calculated to be 2.73×10^3 which is in line with a vesicle morphology.[2]

Transmission Electron Microscopy (TEM) analysis was performed on a JEOL 2100 electron microscope at an acceleration voltage of 200 kV. All samples were diluted with deionized water and then deposited onto formvar-coated copper grids. After roughly 1 min, excess sample was blotted from the grid and the grid stained with an aqueous 1 wt% uranyl acetate (UA) solution for 1 min prior to blotting, drying and microscopic analysis.

Cryogenic Transmission Electron Microscopy (Cryo-TEM) imaging was performed on a JEOL JEM-2100 plus microscope operating at an acceleration voltage of 200 kV. Samples for cryo-TEM were prepared on lacey carbon grids (EM Resolutions). After 200-fold dilution with deionized water, 8 μL of sample were deposited onto the grid followed by blotting for approximately 5 s and plunging into a pool of liquid ethane, cooled using liquid nitrogen in order to vitrify the samples. Then, transfer into a pre-cooled cryo-TEM holder using liquid nitrogen, was performed prior to the microscopic analysis.

Confocal Laser Scanning Microscopy (CLSM) was performed on FV3000 (Olympus) confocal microscope and the 60x oil lens was used for imaging. Images were acquired using the 488 nm (green channel) and the 561 nm (red channel) excitation wavelengths. Freshly prepared and purified solutions of FAM-**H2** (green-emitting dye), TAMRA-**cDNA** (red-emitting dye) and mixed 50% DNA_{14}-$PHPMA_{400}$ copolymer nano-objects at 100-fold dilution were deposited on a glass slide before imaged by CLSM. Images were processed using cellSens (Olympus) and ImageJ image processing software. The LAD-1 LED array driver was purchased from Bio Research Centre Co., Ltd. The array was composed of 96 405 nm LEDs which each output a light power of 20 mW at 13.5 V when measured with an LMP-100 light power sensor (sensor area: 5.5 mm x 4.8 mm), placed directly above an array LED. For the Thermomixer setup, the LED-array was suspended over the sample holder *via* a clamp stand. For the Incubator setup the LED-array was placed upon a shaker plate, face up with a 96-microwell plate placed directly upon it, into which 150 µL eppendorfs containing sample were placed.

Experimental Procedures

Synthesis of 4-cyano-4-[(ethylsulfanylthiocarbonyl)sulfanyl] pentanoic acid (CEPA)

4-Cyano-4-[(ethylsulfanylthiocarbonyl)sulfanyl] pentanoic acid chain transfer agent (CEPA CTA) was synthesized according to a previously described process.[3] Sodium ethanethiolate (10.0 g, 0.119 mol, 1 eq) was suspended in 500 mL of dry diethyl ether at 0 °C. Carbon disulfide (7.74 mL, 0.131 mol, 1.1 eq) was subsequently added dropwise over 10 min, resulting to the formation of a thick yellow precipitate of sodium S-ethyl trithiocarbonate. After 2 h of stirring at room temperature, solid iodine (15.1 g, 0.059 mol, 0.5 eq) was added to the reaction medium. After 2 h, the solution was washed three times with aqueous sodium thiosulfate (1 M), water and finally saturated NaCl solution. The organic layer was thoroughly dried over $MgSO_4$ and the crude bis-(ethylsulfanylthiocarbonyl) disulfide was then isolated by rotary evaporation (16.0g, 0.058 mol, 98%).

A solution of bis-(ethylsulfanylthiocarbonyl) disulfide (16.0 g, 0.058 mol, 1 eq) and 4,4′-azobis(4-cyanopentanoic acid) (ACVA) (24.5 g, 0.087 mol, 1.5 eq) in 500 mL of ethyl acetate was heated at reflux for 18 h under N_2(g) atmosphere. Following rotary evaporation of the solvent, the crude CEPA was isolated by column chromatography using silica gel as the stationary phase and 75:25 DCM-petroleum ether as the eluent. The isolated product was

precipitated out of solution by using hexane leaving a yellow-light orange solid. The final product was collected and dried under reduced pressure to afford pure CEPA CTA (10.95 g, 0.042 mol, 36%). ^{1}H-NMR (400 MHz, $CDCl_3$): δ (ppm) 3.35 (q, 2H, S-CH_2-CH_3), 2.38-2.71 (m, 4H, CH_2-CH_2), 1.89 (s, 3H, C(CN)-CH_3), 1.36 (t, 3H, S-CH_2-CH_3). ^{13}C-NMR (100 MHz, $CDCl_3$): δ (ppm) 216.6 (Cc), 176.9 (Ci), 118.9 (Cf), 46.2 (Cd), 33.5 (Cg), 31.4 (Cb), 29.5 (Ch), 24.9 (Ce), 12.8 (Ca). FT-IR (neat): ν (cm^{-1}) 1709 (C=O), 1073 (C=S)), 810 (C-S). HR-MS: m/z $[C_9H_{13}NO_2S_3+Na]^+$ calc. 286.0001 g mol^{-1}, found 286.0001 g mol^{-1}.

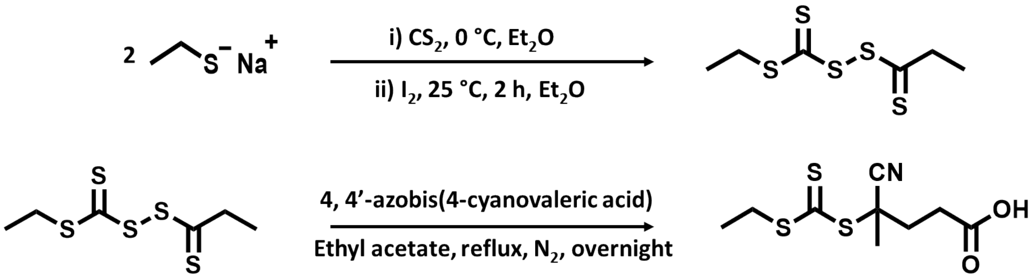

Scheme S1. Synthesis of CEPA-CTA.

Synthesis of poly(ethylene glycol)$_{113}$-CEPA macro-CTA (PEG$_{113}$ macroCTA)

PEG$_{113}$ macroCTA was synthesized according to a previously reported method with slight modification.[4] Poly(ethylene glycol) methyl ether (average M_n=5,000 g mol^{-1}, PEG$_{113}$-OH) (4.75 g, 0.98 mmol, 1 eq) was dissolved in 150 mL of dry DCM. The resulting solution was then purged with N_2(g) for 30 min. After complete dissolution, CEPA CTA (1 g, 3.8 mmol, 4 eq), DCC (392 mg, 1.9 mmol, 2 eq) and DMAP (23 mg, 0.19 mmol, 0.2 eq) were added to the reaction mixture. The esterification reaction proceeded with stirring at room temperature for 18 h under continuous N_2(g) flow. After this period, further DCC (392 mg, 1.9 mmol, 2 eq) and DMAP (23 mg, 0.19 mmol, 0.2 eq) were added to the reaction mixture and then stirred at room temperature for an additional period of 6 h under continuous N_2(g) flow. The solution was then filtered to remove unreacted DCC and DMAP. The product was collected by 5 times of precipitation using cold diethyl ether as non-solvent, redissolved in deionized water and dialyzed against nanopure water using a 1,000 kDa MWCO membrane for 1 day (yield = 58%). The received PEG$_{113}$ macroCTA solution was lyophilized to give a light yellow powder as the final product (2.90 g, 0.55 mmol, 58%).^{1}H-NMR (400 MHz, $CDCl_3$): δ (ppm) 4.25 (m, 2H, CO_2-C$\boldsymbol{H_2}$), 3.44-3.82 (m, 2H, C$\boldsymbol{H_2}$O), 3.36 (s, 3H, O-C$\boldsymbol{H_3}$), 3.34 (q, 2H, CH_3-C$\boldsymbol{H_2}$), 2.64 (m, 2H, C$\boldsymbol{H_2}$-CO_2), 2.34-2.56 (m, 2H, C(CN)-C$\boldsymbol{H_2}$), 1.87 (s, 3H,

C$\boldsymbol{H_3}$-C(CN)), 1.35 (t, 3H, C$\boldsymbol{H_3}$-CH_2). SEC (5 mM NH_4BF_4 in DMF, λ = 309 nm) M_n = 4.97 kg mol^{-1}, $Đ_M$ = 1.17.

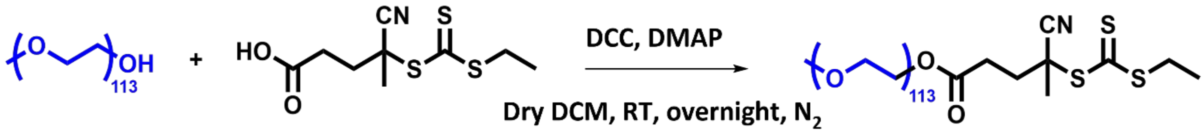

Scheme S2. Synthesis of PEG_{113} macroCTA.

Synthesis of $ssDNA_{14}$ macroCTA by solution approach

Amine-modified oligonucleotide ($ssDNA_{14}$-NH_2) (1 mM, 1 µl, 1 eq.) was mixed with borate buffer pH 7.5 (45 µl), then mixed with CEPA (0.5 M, 20 µL, 500 eq.), EDC·HCl (1 M, 10 µl, 500 eq), NHS (1 M, 10 µl, 500 eq), and DIPEA (1 M, 10 µl, 500 eq) in DMF at 21 °C 400 rpm overnight. Residual chemicals were removed *via* ethanol precipitation by adding 300 vol% ethanol to the reaction mixture and incubating the samples at -20 °C for 6 hours. After centrifugation at 15000 rpm for 30 min at 4 °C the supernatant was removed, and the DNA pellet washed with ice-cold 70% ethanol followed by a repeated centrifugations using the same settings as described above. The supernatant of the washing solution was removed and the DNA pellet was dried on air.

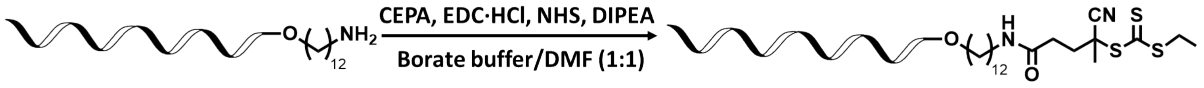

Scheme S3. Synthesis of $ssDNA_{14}$ macroCTA by solution approach.

Synthesis of $ssDNA_{14}$ macroCTA by solid support approach

250 µL of DEAE Sepharose suspension was used as solid support and pipetted into an empty Glen Research column housing and washed with 20 ml of H_2O followed by 12 ml of DEAE binding buffer (10 mM acetic acid and 0.005% Triton X-100) using a syringe. The DNA-NH_2 (10 µM, 1 ml, 1 eq) was loaded onto the column after dissolving in 1 ml of DEAE binding buffer. The column was then washed with 3 ml of DEAE binding buffer, followed by 1 mL of H_2O and 4 ml of DMF to switch the solvent system from water to DMF. At least 50

nmol of oligonucleotide can be loaded onto one 250-µL DEAE Sepharose column. The activated ester solution was composed of CEPA (0.5 M, 100 µL, 500 eq), EDC·HCl (1M, 50 µL. 500 eq), NHS (1 M, 50 µL. 500 eq), and DIPEA (1 M, 50 µL. 500 eq) in solvent (DMF) and was incubated at 21 °C for 30 min before use. The CEPA active ester solution was loaded onto the solid support column in 1 ml of DEAE bind buffer and incubated for 10 min. After the reaction was completed, the column was washed with 4 ml of the reaction solvent (DMF) followed by 3-5 ml of DEAE binding buffer. Finally, the DNA was eluted with 4 ml of DEAE elution buffer (1.5 M NaCl, 50 mM Tris-HCl [pH 8.0], and 0.005% Triton X-100) using a syringe. Product formation was verified via LC-MS: m/z [C159H211N50O91P14S3--H] calc. 4807.900 g mol-1, found 4807.314 g mol^{-1}.

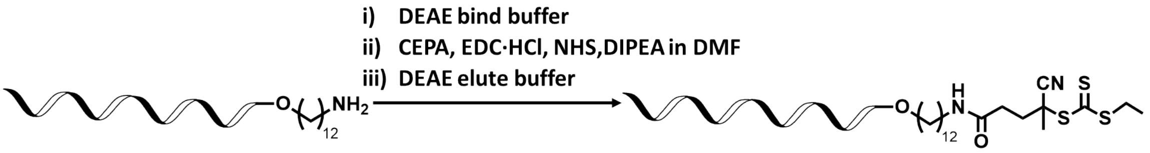

Scheme S4. Synthesis of ssDNA$_{14}$ macroCTA by solid support approach.

Synthesis of DNA−Polymer Conjugates by Photopolymerization-Induced Self Assembly

ssDNA$_{14}$-macroCTA (250.5 µL, 20 mg/mL, 1 eq) was added in a centrifuge tube containing HPMA (2.5 mg for 5%w/w, 5 mg for 10%w/w, 200,300,400 eq). Then, glucose solution (6 µL, 0.84 M), nanopure water (8.4 µL) and GOx solution (8 µL, 12.5 µM) were added into the mixing vial, respectively, resulting in a total volume of 50 μL. The mixture was shaken via vortexer to produce a clear colourless solution which was then transferred to pointed base PCR plate. Mineral oil was added around 200 µL on top of the mixture and the plate was covered by plate seal, and placed in LED array setup, which was contain in an incubator to maintain a temperature of 37 °C. The solution was then exposed to 405 nm light for 2 h, resulting in the solution turning opaque and milky white.

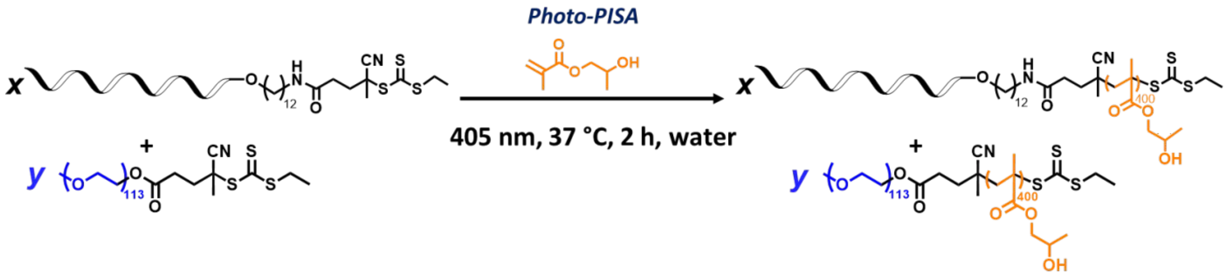

Scheme S5. Synthesis of DNA−Polymer Conjugates by Photopolymerization-Induced Self Assembly.

In order to study the DNA hybridization of the particle surface, a 10 mM $MgCl_2$ in 1×TAE solution was used as a buffer to provide optimal DNA hybridization conditions. The surface hybridisation of nanoobjects formed using different ratios of ssDNA and PHPMA containing diblock copolymers was investigated. Nanopure water (177 or 159 μL) was added into $MgCl_2$ (2 μL, 1M), nanoobject solution (1 μL, 100 mg/ml), TAE (20 μL, 10×) to prepare the particle solution under buffered condition (10 mM) in 1.5 mL tube. The tube was shaken at room temperature at least 30 min and then transferred to a DLS microcuvette. DLS was used to investigate particle size and size dispersity. Subsequently, varying systematic amounts of cDNA (100 μM) was added to the centrifuge tubes, containing the nanoobjects with varying ratios of ssDNA. The centrifuge tube was shaken at room temperature for at least 30 min before investigation by DLS, TEM and confocal microscopy.

Hybridization chain reaction (HCR) study

The HCR reaction was performed under the same conditions for the free ssDNA (SC) and for the ssDNA containing nanoobject systems. Before performing HCR, hairpin1 (**H1**) (50 μM, 5 μL) and hairpin2 (**H2**) (50 μM, 5 μL) were incubated at 95 °C for 2 min then quenched at -20 °C for 1 min. The initiator (**I**) (0.2 eq), **H1** (varied eq.), and **H2** (varied eq.) were added to the ssDNA (SC) or ssDNA containing nanoobject systems (10 μM, 1 eq) under 10 mM $MgCl_2$ in 1×TAE condition in a 1.5 mL tube at room temperature for 30 min. The ssDNA samples were analyzed by native PAGE. ssDNA containing nanoobject systems were also analysed by DLS, TEM, Cryo-TEM and confocal microscopy.

Table S1. List of ssDNA-copolymer nanoparticles obtained from RAFT aqueous dispersion Photopolymerization-Induced Self Assembly and Summary of Characterization Data

Sample	Targeted DP	Actual DP[a]	Conversion[a] (%)	M_n [b] (kg·mol^{-1})	$Đ_M$[b]	D_h[c] (nm)	PD[c]	Morphology[d]
DNA – $PHPMA_{400}$	400	320	~80	123	1.5	90	0.12	S
90% DNA–$PHPMA_{400}$	400	288	~72	108	1.9	219	0.42	S+LR+V
50%DNA– $PHPMA_{400}$	400	385	~96	137	1.5	154	0.04	V
10%DNA]-$PHPMA_{400}$	400	356	~89	54	1.5	186	0.09	V
PEG_{113}– $PHPMA_{400}$	400	352	~88	68	1.4	459	0.03	V

[a] Calculated from ^{1}H NMR spectroscopy (400 MHz) in deuterated MeOD.

[b] Determined by DMF SEC with poly(methyl methacrylate) (PMMA) standards.

[c] Determined by DLS using z-average data.

[d] Determined by TEM. S = Spheres, LR = Lumpy Rod, and V = Vesicle.

Table S2. DNA sequences

Name	DNA sequence (5'- 3')	Extinction coefficient (L (mole·cm)$^{-1}$)
$ssDNA_{14}$ (SC)	TGTAGCGTTGTTGC	128,400
$ssDNA_{14}$-NH_2	/5AmMC12/ TGTAGCGTTGTTGC	128,400
cDNA	GCAACAACGCTACA	139,600
cDNA-TAMRA	/56-TAMN/GCAACAACGCTACA	168,700
ncDNA	TGTAGCGTTGTTGC	128,400
I	GAGGAGGGCAGCAAACGGGAAGAG TCTTCCTTTACGGCAACAACGCTACA	496,900
I-TAMRA	/56-TAMN/ GAGGAGGGCAGCAAACGGGAAGAGTCTTC CTTTACGGCAACAACGCTACA	526,000
H1	CGTAAAGGAAGACTCTTCCCGTTTG CTGCCCTCCTCGCATTCTTTCTTGAGGAGG GCAGCAAACGGGAAGAG	677,600
H2	GAGGAGGGCAGCAAACGGGAAGAGTCTTC CTTTACGCTCTTCCCGTTTGCTGCCCTCCTC AAGAAAGAATGC	678,200
H2-FAM	/FAM/GAGGAGGGCAGCAAACGGGAAGAGT CTTCCTTTACGCTCTTCCCGTTTGCTGCCCT CCTCAAGAAAGAATGC	688,200

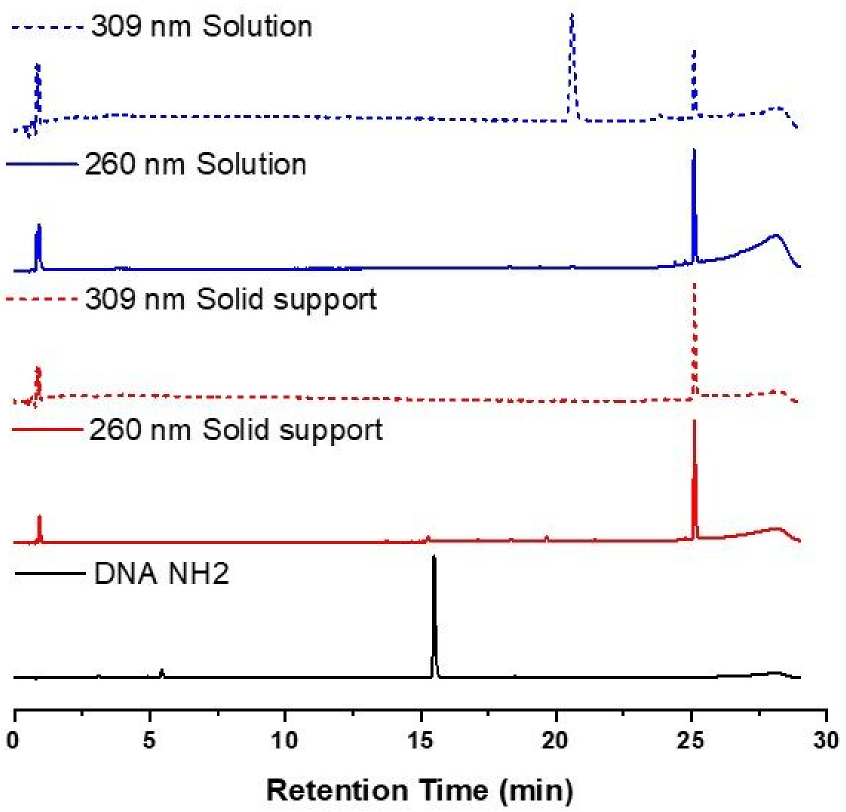

Figure S1. RP-HPLC chromatograms of DNA macroCTA synthesized by solution method (blue line), solid support method (red line) using NH_2-ssDNA as a starting material (black solid line). The chromatograms were investigated at detector wavelength 309 nm (dash line) and detector wavelength 260 nm (solid line). Products eluted with a gradient of buffer A, 0.1 M triethylammonium acetate (TEAA), in a 95:5 mixture of H_2O and acetonitrile and buffer B, 0.1 M TEAA, in a 30:70 mixture of H_2O and acetonitrile.

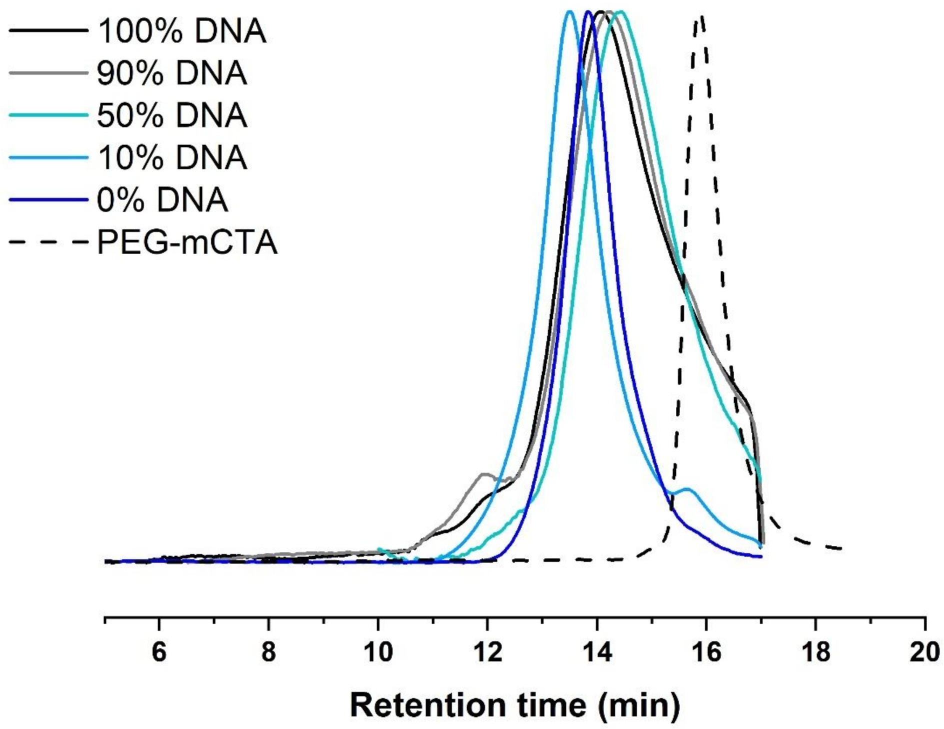

Figure S2. GPC traces of PEG-macroCTA (dot line) and the DNA/PEG polymer conjugates (solid lines) as measured by DMF GPC using polymethylmethacrylate (PMMA) calibration standards.

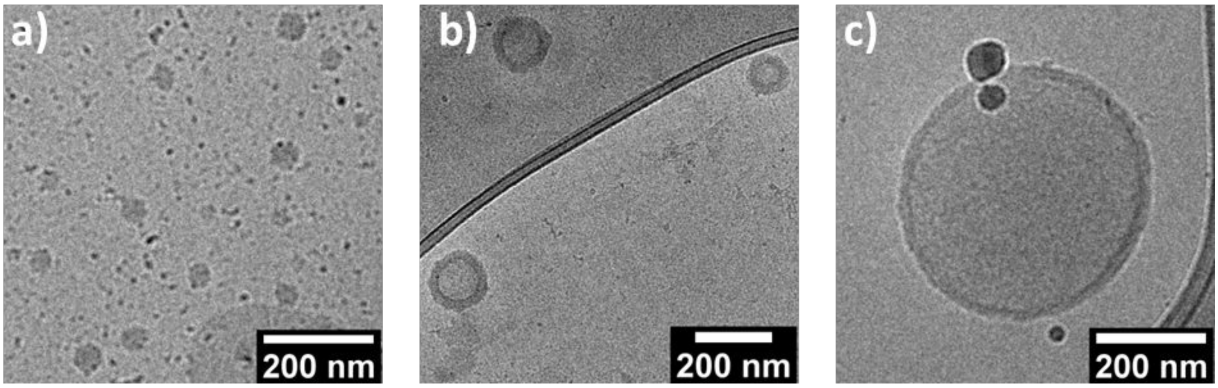

Figure S3. Cryogenic transmission electron microscopy (Cryo-TEM) images of a) DNA – $PHPMA_{400}$, b) 50%$ssDNA_{14}$ – $PHPMA_{400}$, and c) PEG_{113}– $PHPMA_{400}$ assign the copolymer morphology.

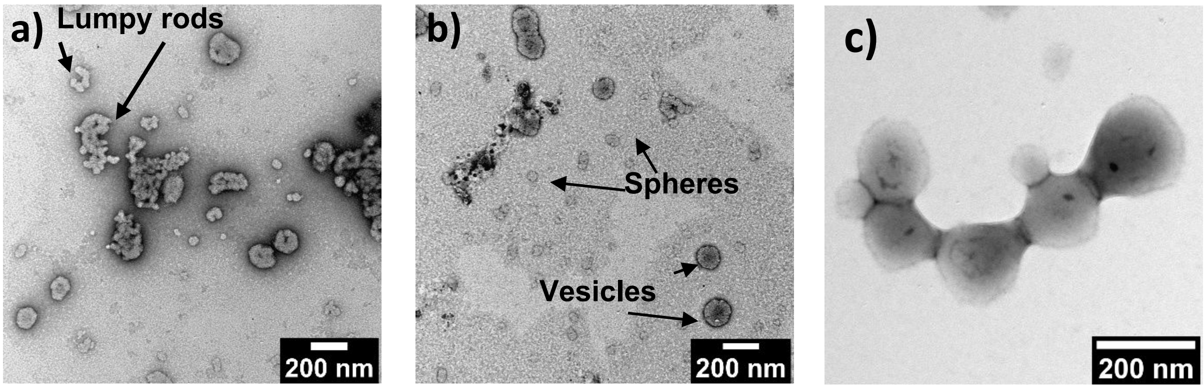

Figure S4. Transmission electron microscopy (Cryo-TEM) images of a) and b) 90%DNA – $PHPMA_{400}$ and b) 10% DNA – $PHPMA_{400}$ diblock copolymer.

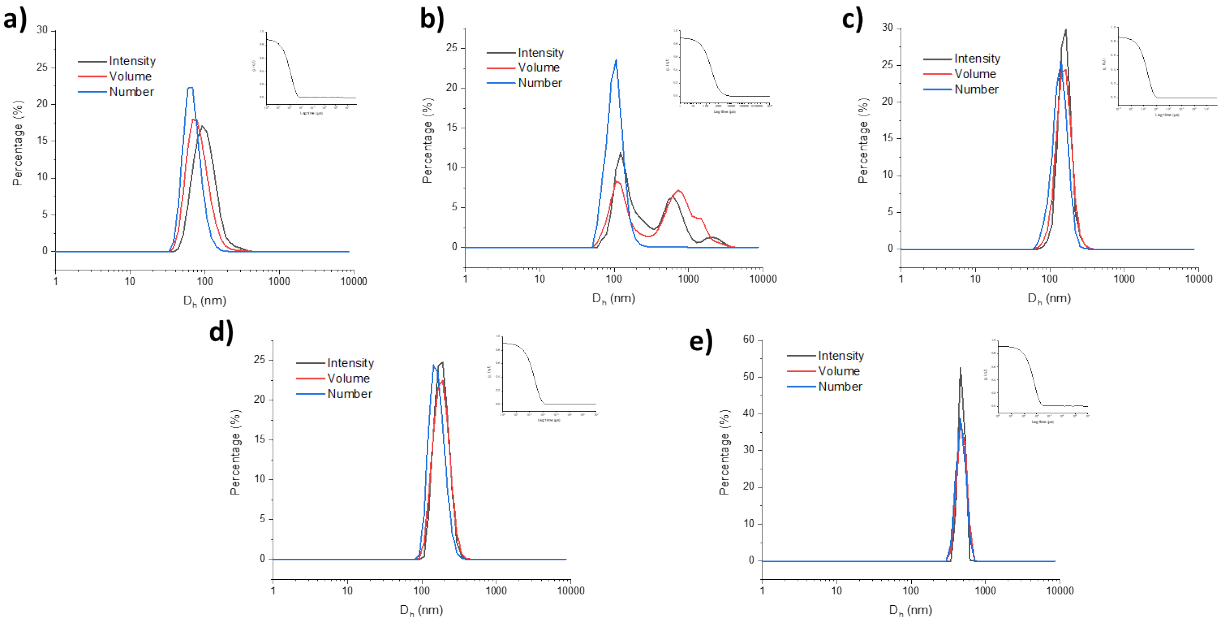

Figure S5. Dynamic light scattering (DLS) of mixed DNA and PEG based objects with different percentages of DNA in the corona a) 100% DNA, b) 90% DNA, c) 50% DNA, d) 10% DNA, and e) 0% DNA.

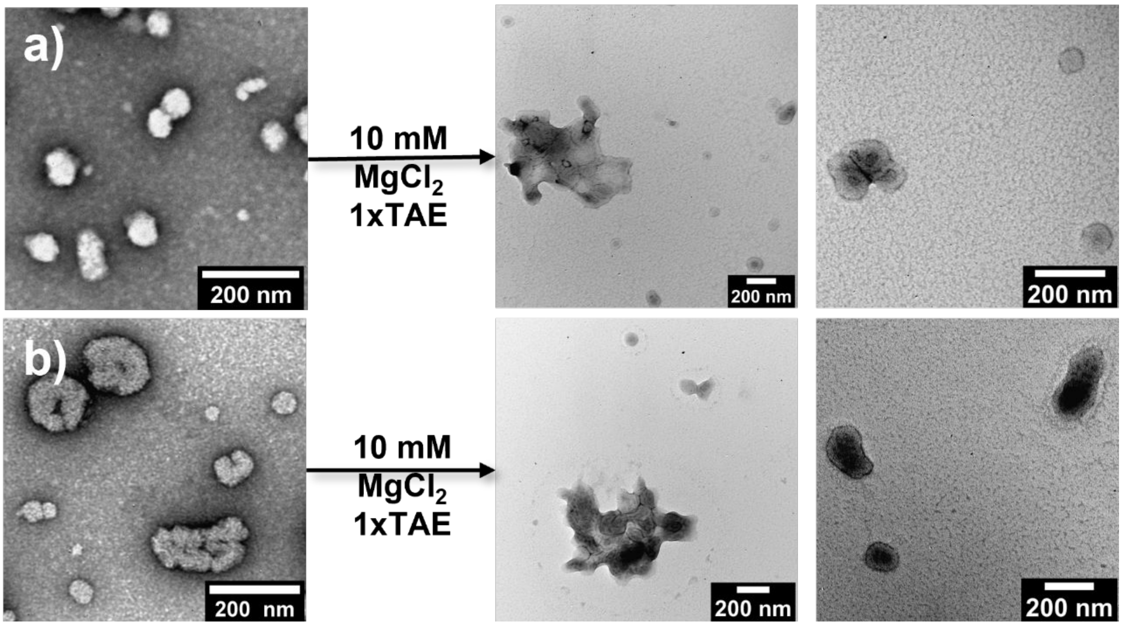

Figure S6. TEM images of a) DNA-PHPMA$_{400}$ particles and b) 50%DNA-PHPMA$_{400}$ particles aggregated when salt/TAE buffer was added.

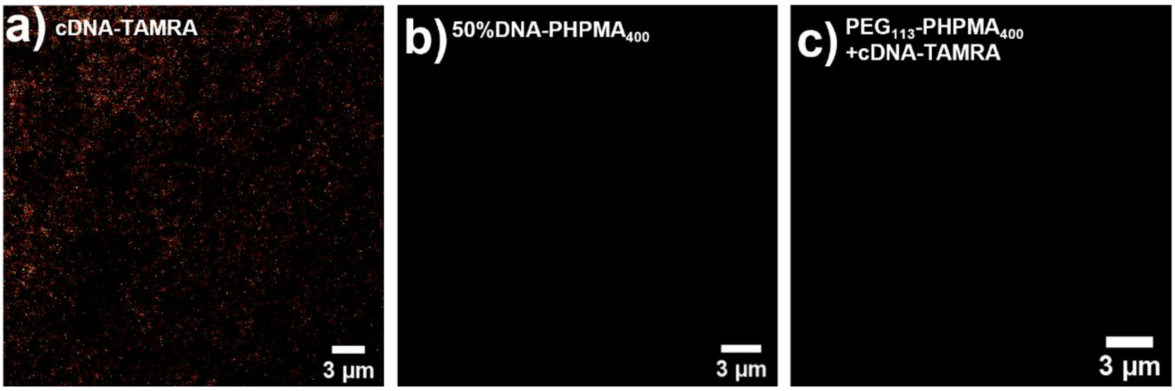

Figure S7. **C**onfocal images of hybridization studying which a) TAMRA-cDNA, b) 50%DNA-PHPMA$_{400}$, and c) PEG$_{113}$-PHPMA$_{400}$+ TAMRA-cDNA.

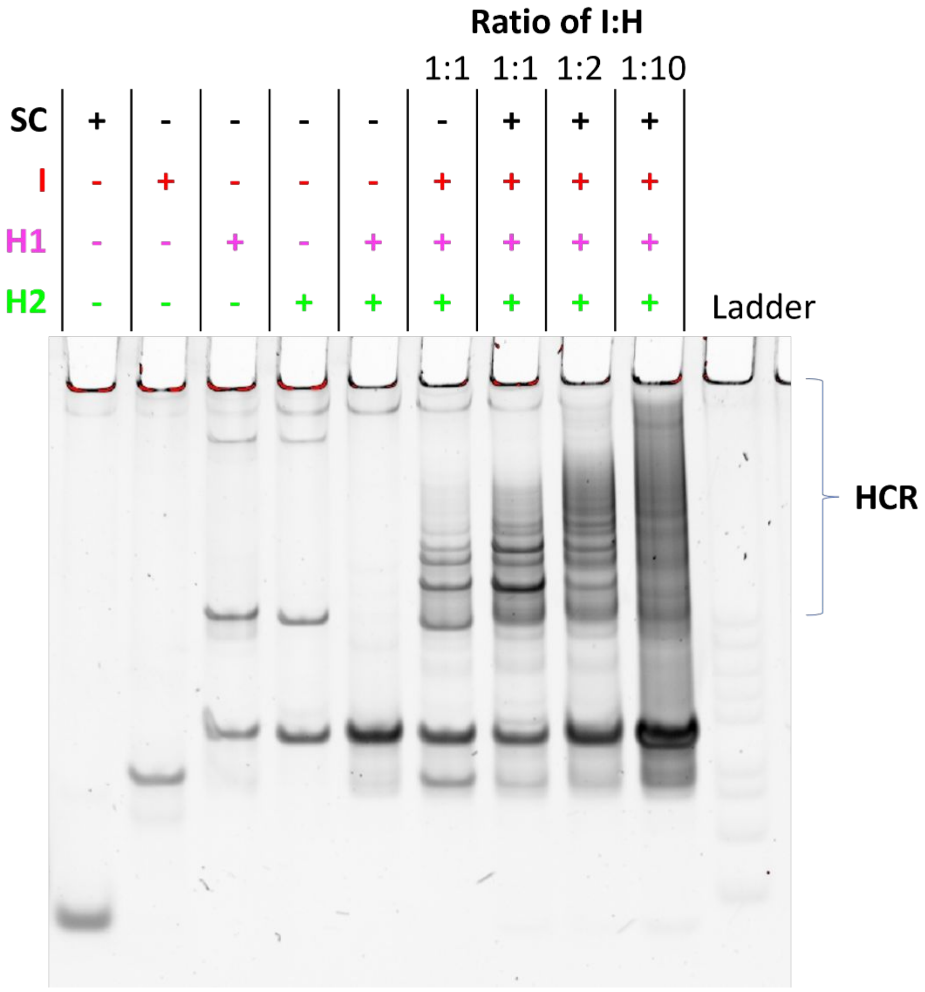

Figure S8. Native poly(acrylamide) gel electrophoresis (10%) of hybridization chain reaction (HCR) study utilizing the ssDNA (SC). The ratios of initiator's concentration to hairpin DNAs' concentration (**I**:**H**) were 1:1, 1:2, and 1:10 with a constant final concentration of 5 µM hairpin DNAs. **H1** and **H2** do not hybridize before triggered by **I**, so no reaction is observed (lane 5). HCR products are only formed when **I** is present (lane 6). Effect of **I**:**H** ratio on HCR amplification (Lanes 7–9): three different ratios of **I**:**H** (1:1, 1:2, and 1:10) in a 5 µM mixture of **ssDNA(SC)**, **I**, **H1** and **H2** were tested. Before mixing, **H1** (50 µM, 10 µL) and **H2** (50 µM, 10 µL) were incubated at 95 °C for 2 min then quenched at -20 °C for 1 min.

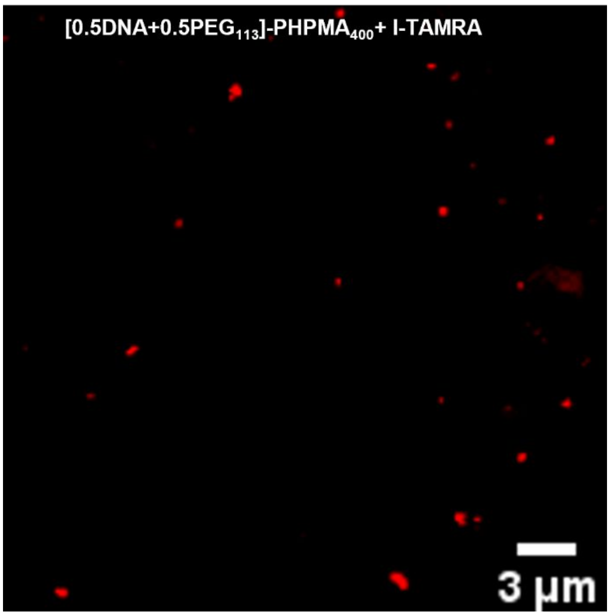

Figure S9. Confocal images of hybridization chain reaction studying of 50%DNA - PHPMA$_{400}$ + I-TAMRA.

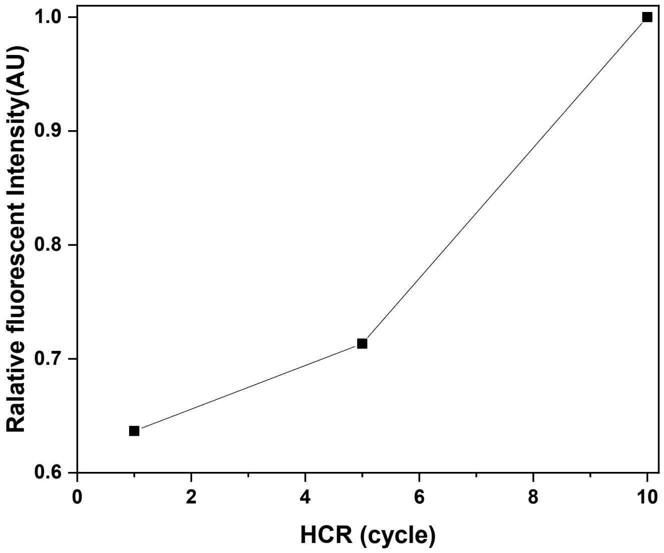

Figure S10. Fluorescence intensity analysis at different HCR Cycles. The presented values were calculated for each particle using the CLSM images from Figure 3g-j, obtained through ImageJ analysis of the CLSM images in the green channel.

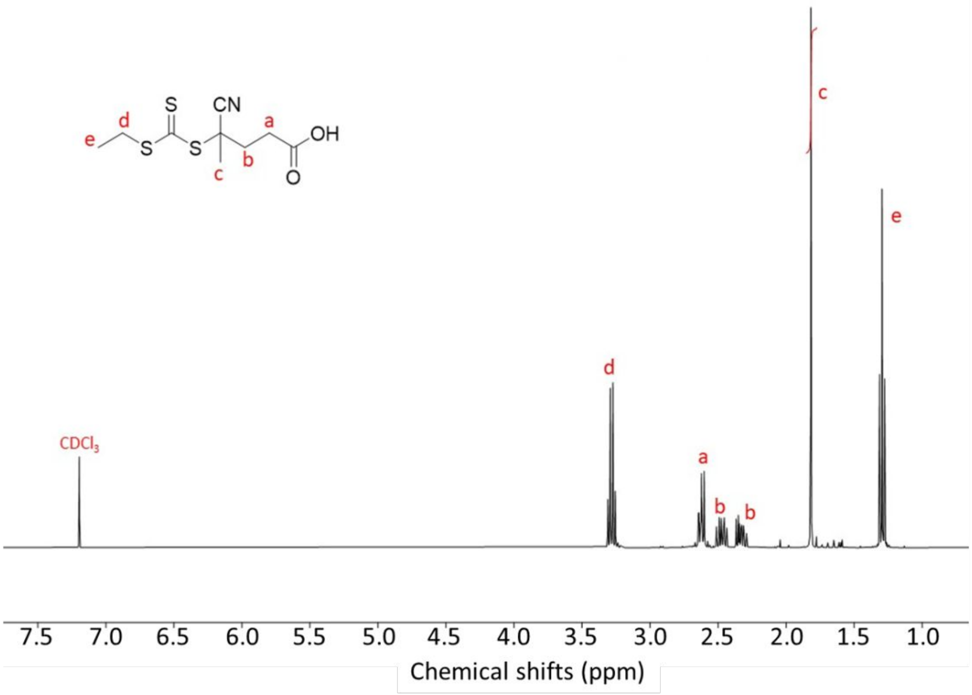

Figure S11. ^{1}H-NMR spectrum of CEPA-CTA in $CDCl_3$.

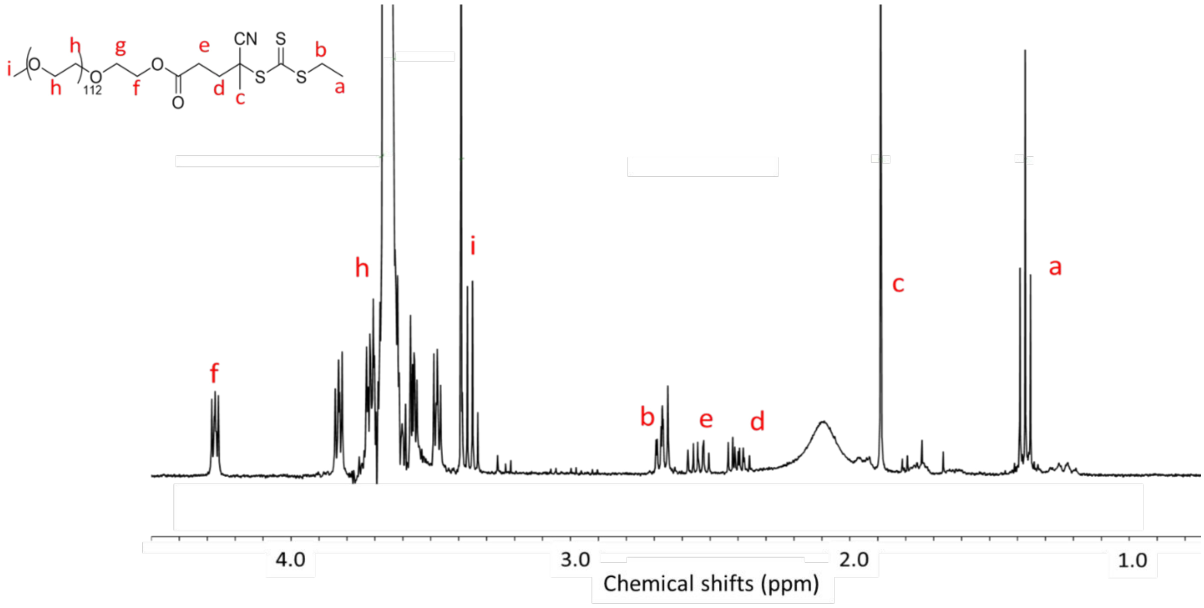

Figure S12. ^{1}H-NMR spectrum of PEG_{113}-macroCTA in $CDCl_3$.

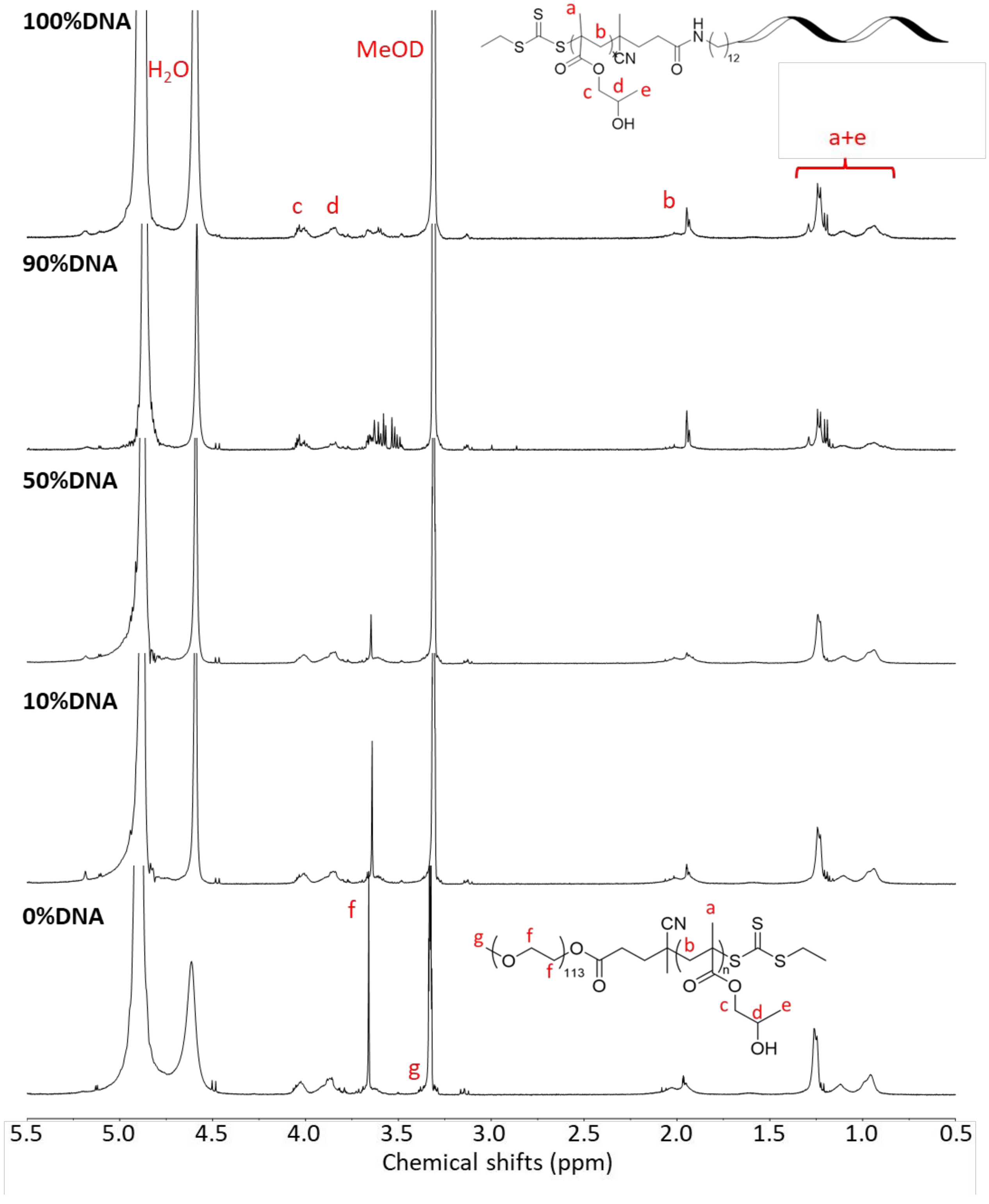

Figure S13. ^{1}H-NMR spectra of different mole fractions of DNA/PEG polymer conjugates in MeOD

References

1. Andersson, M.; Wittgren, B.; Wahlund, K.-G., Accuracy in Multiangle Light Scattering Measurements for Molar Mass and Radius Estimations. Model Calculations and Experiments. *Analytical Chemistry* **2003,** *75* (16), 4279-4291.
2. Patterson, J. P.; Robin, M. P.; Chassenieux, C.; Colombani, O.; O'Reilly, R. K., The analysis of solution self-assembled polymeric nanomaterials. *Chem Soc Rev* **2014,** *43* (8), 2412-25.
3. Johnson, R. N.; Burke, R. S.; Convertine, A. J.; Hoffman, A. S.; Stayton, P. S.; Pun, S. H., Synthesis of statistical copolymers containing multiple functional peptides for nucleic Acid delivery. *Biomacromolecules* **2010,** *11* (11), 3007-3013.
4. Tan, J.; Sun, H.; Yu, M.; Sumerlin, B. S.; Zhang, L., Photo-PISA: Shedding Light on Polymerization-Induced Self-Assembly. *ACS Macro Letters* **2015,** *4* (11), 1249-1253.
